# Adult Cardiac Stem Cell Aging: A Reversible Stochastic Phenomenon?

**DOI:** 10.1155/2019/5813147

**Published:** 2019-02-07

**Authors:** Eleonora Cianflone, Michele Torella, Cristina Chimenti, Antonella De Angelis, Antonio P. Beltrami, Konrad Urbanek, Marcello Rota, Daniele Torella

**Affiliations:** ^1^Molecular and Cellular Cardiology, Department of Medical and Surgical Sciences, Magna Graecia University, Catanzaro 88100, Italy; ^2^Department of Physiology, New York Medical College, Valhalla, New York, USA; ^3^Department of Cardiothoracic Sciences, University of Campania “L. Vanvitelli”, Naples, Italy; ^4^Department of Cardiovascular, Respiratory, Nephrologic, Anesthesiologic and Geriatric Sciences, Sapienza University of Rome, Rome 00161, Italy; ^5^Department of Experimental Medicine, Section of Pharmacology, University of Campania “L. Vanvitelli”, Naples 80121, Italy; ^6^Department of Medicine, University of Udine, Udine, Italy

## Abstract

Aging is by far the dominant risk factor for the development of cardiovascular diseases, whose prevalence dramatically increases with increasing age reaching epidemic proportions. In the elderly, pathologic cellular and molecular changes in cardiac tissue homeostasis and response to injury result in progressive deteriorations in the structure and function of the heart. Although the phenotypes of cardiac aging have been the subject of intense study, the recent discovery that cardiac homeostasis during mammalian lifespan is maintained and regulated by regenerative events associated with endogenous cardiac stem cell (CSC) activation has produced a crucial reconsideration of the biology of the adult and aged mammalian myocardium. The classical notion of the adult heart as a static organ, in terms of cell turnover and renewal, has now been replaced by a dynamic model in which cardiac cells continuously die and are then replaced by CSC progeny differentiation. However, CSCs are not immortal. They undergo cellular senescence characterized by increased ROS production and oxidative stress and loss of telomere/telomerase integrity in response to a variety of physiological and pathological demands with aging. Nevertheless, the old myocardium preserves an endogenous functionally competent CSC cohort which appears to be resistant to the senescent phenotype occurring with aging. The latter envisions the phenomenon of CSC ageing as a result of a stochastic and therefore reversible cell autonomous process. However, CSC aging could be a programmed cell cycle-dependent process, which affects all or most of the endogenous CSC population. The latter would infer that the loss of CSC regenerative capacity with aging is an inevitable phenomenon that cannot be rescued by stimulating their growth, which would only speed their progressive exhaustion. The resolution of these two biological views will be crucial to design and develop effective CSC-based interventions to counteract cardiac aging not only improving health span of the elderly but also extending lifespan by delaying cardiovascular disease-related deaths.

## 1. Introduction

Over the last decades, average life expectancy has significantly increased worldwide although several chronic diseases continue to grow, with aging as their main risk factor [1]. Aging is a natural and inevitable degenerative process of biological functions characterized by the progressive decline in tissue and organ homeostasis and function. Despite the significant improvements in diagnosis and treatment, the majority of individuals older than 65 years of age suffer from an elevated risk to develop cardiovascular diseases (CVDs), with a decline in the quality of life and in the ability to perform the normal activities of daily living [1]. Aging produces numerous changes in the human heart at structural, molecular, and functional levels [2]. The most significant age-related alterations in the heart are left ventricular (LV) hypertrophy, fibrosis, denervation, and maladaptive remodelling that most frequently lead to diastolic dysfunction and heart failure with preserved ejection fraction [2, 3].

Nowadays, one of the central aims of cardiovascular research is to uncover the mechanisms that lead to the age-associated CVDs. One of the most studied phenomena occurring with aging is the change in the redox state occurring between the embryonic life and the postnatal life whereby similar metabolic changes have been found then to occur in the progression from the adult to the aged myocardium. During the embryonic life and the foetal life, cardiomyocyte (CM) formation and proliferation are the main mechanisms underlying cardiac contractile muscle development. The latter process takes place in a hypoxic environment characterized by a low reactive oxygen species (ROS) levels and by an anaerobic metabolism, which are the major energy source for myocardial cell maintenance [4]. Postnatal normoxia increases ROS levels producing oxidative stress that leads to cell cycle exit and terminal differentiation of CMs [5]. In the adult heart, oxidative stress induced by normoxia can further modulate cardiac function causing overtime heart decompensation [6]. Thus, the oxidative state and cell metabolism have been recognized as important determining factors for cell fate and cell cycle status in the heart [6].

The inevitable decline of life with aging has been related to two pivotal mechanisms: an aging telomere-dependent phenomenon that leads to telomere attrition and an aging telomere-independent process. The latter that anyway may also result in telomere attrition is secondary to the alteration in the intracellular redox state and promotion of oxidative modification of regulatory molecules and contractile proteins [7, 8]. Particularly, in the heart, the oxidative stress directly affects cardiomyocyte (CM) contraction [7, 8] leading to altered cellular homeostasis that finally promotes a progressive cardiac dysfunction. This condition fosters the development of an aging cardiac myopathy characterized by changes in the microenvironment and the stimuli on the aged myocardium while the number of CMs decreases as a function of age [9–12]. In order to compensate for the age-related modifications, the myocardium increases its muscle mass by CM hypertrophy, which in the long term however results in a weakened cardiac function and in fibroblast proliferation causing myocardial and arterial fibrosis. This prototypical pathologic cardiac remodelling produces an increase in supraventricular and ventricular arrhythmias [13], and it also produces a further increase of ROS, a characteristic of the aged organs [14]. Indeed, ROS are considered a risk factor for a wide range of diseases in elderly and their role has been continuously investigated in these years in the field of cardiac regenerative medicine with the aim to develop applicable protocols to attenuate their formation and to delay the onset of cardiac morbidity in the elderly [15].

**Figure 1:**
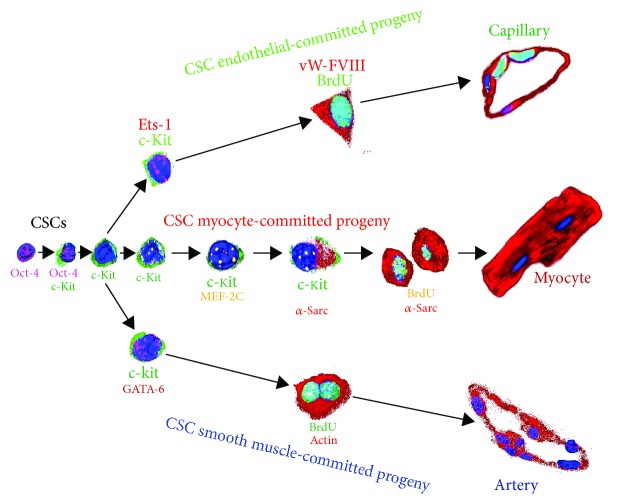
Schematic representation of the transitional sequence of cardiac stem cell-committed progeny. Quiescent, primitive, undifferentiated cardiac stem cells express Oct-4 (pink fluorescence), become activated, and start expressing c-kit (green fluorescence). In response to stress, these cells multiply and lose expression of Oct-4. The resulting c-kit pos/Oct-4 neg cells are still uncommitted to one specific cardiac cell lineage. After further expansion and differentiation, the cells induce expression of transcription factors specific to one cardiac lineage (GATA4, ETS1, or GATA6) and differentiate into one of the three cardiac cell types—cardiomyocytes, endothelial, and smooth muscle cells—respectively. These newly formed cardiac cells can undergo a few rounds of replication before becoming terminally differentiated. CSC: cardiac stem cell; vWF-VIII: von Willebrand factor VIII. Figure 1 is reproduced from Georgina M. Ellison et al., (under the Creative Commons Attribution License/public domain) [54].

Despite that, for almost a century and until the new millennium, the adult heart was considered a postmitotic organ, several recent studies have supported the notion that it possesses a population of endogenous cardiac stem/progenitor cells (CSCs) supporting myocardial cell turnover and regeneration due to their intrinsic potential to differentiate in all cardiac cell lineages [16–23] (Figure 1). This discovery opened a new era for myocardial regeneration where endogenous cardiac stem/progenitor cells were introduced as direct regenerative agents and/or endogenous targets of regenerative therapy to effectively replenish the heart muscle cells, lost by injury and/or age, in order to improve/normalize myocardial function [16]. However, the pathological and pathophysiological cardiomyopathy that occurs with age also affects the stem cell microenvironment modifying adult stem cell biology and then their ability, during lifespan, to repair damaged tissues and organs [24–27]. Accordingly, as shown for other stem cell types [26, 28–31], cardiac stem cell (CSC) potential has also been found to be compromised or even lost with aging as a consequence of the accumulation and activation of senescence factors affecting myocardial homeostasis, producing DNA damage and alteration of the telomere-telomerase system eventually leading to a senescent phenotype of CSCs [32–34]. Despite these evidences, interesting studies have demonstrated that the old decompensated heart appears to maintain a functionally competent pool of CSCs during life and that the senescent phenotype of CSCs may be therefore reverted using growth factors or cardioprotective molecules [35–37]. This accumulating knowledge is fundamental for the prospects of CSCs as main agents for myocardial regeneration because the majority of the patients in need of such therapy are indeed aged subjects.

On this basis, in this review, we will summarize the biological role of endogenous cardiac stem cells and their importance in the cardiac tissue maintenance during mammalian life. We will discuss CSC adaptations and changes in the aged myocardium and the ability of high-reactivity small molecules to diminish the regenerative potential of the resident CSC pool inducing a forced entry in a senescent state with a consequent severe deficit of functionally competent CSCs with time. We finally will discuss several potential molecular mechanisms implicated in the preservation of a competent CSC pool during the lifespan, which could be essential to foster CSC rejuvenation.

Currently, it is well known that ROS signalling is important in the metabolism of embryonic and adult stem cells and may impact stem cell epigenome and their cell fate [38–40] but the exact mechanisms of metabolic regulation of stem cell epigenetics remains still unknown. The importance of redox signalling in the turnover of adult CSCs is nonetheless one of the less-explored areas in cardiac regeneration. As stem cells are considered the most promising tool for regenerative medicine, to shed light in this crucial area is a stimulating avenue for future studies, which will be pivotal to develop new strategies for effective cardiac regeneration medicine with the aim to prolong the length and quality of human lifespan.

## 2. Cardiac Tissue Homeostasis Is Maintained by Endogenous CSC Activation

For many years, cardiovascular diseases, secondary to age-associated changes in the cardiac tissue, have been viewed under the prevalent dogma of the adult heart as a postmitotic organ composed by a predetermined number of CMs established at birth and largely preserved throughout life of the organism until its death [41, 42]. Cardiac growth that occurs postnatally has been then explained solely by CM hypertrophy whereby CMs progressively age and enlarge according with the age of the organism. Based on this premise, cardiac aging has been considered an autonomous process leading with time to molecular modifications of the adult CMs that profoundly alter the characteristics and performance of the heart resulting in a failing cardiomyopathy [42]. However, this static notion of the adult heart has been recently questioned by the evidence that, although age is the major risk and causative factor of the functional maladaptation of the old heart, the chronological age and physical age do not coincide [43]. Thus, the organism age and organ age do not proceed at the same rate [43]. Indeed, in the last decade, it has been reproducibly demonstrated that the adult mammalian heart maintains a cell turnover during the organismal life which includes CM replacement and that the adult heart is able to regenerate after injury through the activation of the endogenous pool of CSCs [16–23, 35, 37, 44]. This new view has led to the reconsideration of the mechanisms implicated in the different manifestations of the aging myopathy with the aim to elucidate CSC contribution in the homeostasis and in the aging of the heart. Accordingly, it has been nowadays largely accepted that the normal homeostasis of the adult heart is balanced by CM death and regeneration events. In the young heart, the generation of new CMs contributes significantly to the normal growth and predominates cell death; on the other hand, in late adulthood, CM death predominates the formation of new CMs [37]. However, during cardiac development and maturation, also the newly formed CMs in the adult life become terminally differentiated cells after a few rounds of proliferation; the latter further supports a fundamental role for CSCs in the maintenance of normal cardiac cellular homeostasis throughout the life of the organism [45].

Adult stem cells, in the course of the mammalian life, persist mainly in a strictly quiescent state, a property that is crucial for their self-renewal capacity [46–49]. Despite their quiescence, adult stem cells are empowered with an intrinsic potential to quickly gain cell cycle competence and to proliferate in order to regenerate their specific tissue, within a limited time, in response to damage or stress. This requires metabolic plasticity in order to adapt to either quiescence or highly proliferative state.

**Figure 2:**
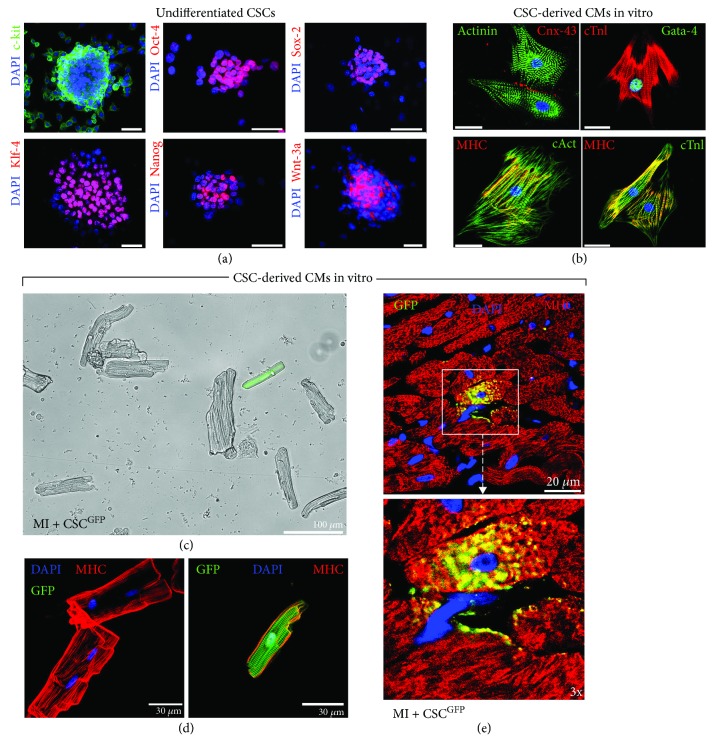
c-Kit^pos^ CSCs are multipotent *in vitro* and *in vivo*. (a) Undifferentiated c-kit^pos^ (green) CSC-derived cardiospheres express multipotent stemness markers (c-kit, Oct-4, Sox-2, Klf-4, and Nanog) and Wnt3a (red). Bar = 50 *μ*m. (b) CSC-derived contracting CMs *in vitro* express contractile proteins (actinin, cTnI, MHC, and cardiac actin) with coexpression of cardiac transcription factor (Gata-4). The CSC-derived CMs exhibit well-defined sarcomeric structures (z lines and dots) as well as gap junction formation (Cnx-43) between cells. DAPI stain nuclei are in blue. Bar = 20 *μ*m. (c) Light microscopy image of freshly isolated adult cardiomyocytes from a dissociated rat heart 28 days after myocardial infarction (MI) and CSC GFP injection (MI + CSC GFP) shows a CSC-derived GFP-positive cardiomyocyte. (d) Confocal microscopy images show host-derived preexisting GFP^neg^ cardiomyocytes as compared to CSC-derived GFP^pos^ cardiomyocytes isolated from MI + CSC GFP rat hearts at 28 days after MI. Note that GFP^pos^ cardiomyocytes are of smaller size and mononucleated when compared to surviving binucleated GFP^neg^ cardiomyocytes of the host. (e) Representative confocal images show at high magnification a CSC-derived newly formed GFP^pos^ cardiomyocyte in the infarct border zone 28 days after MI treated with CSC GFP. Figure 2 is reproduced from Carla Vicinanza et al., (under the Creative Commons Attribution License/public domain) [55].

The embryonic, neonatal, and adult mammalian heart possesses, dispersed throughout its interstitium, the so-called cardiac stem cells (CSCs) and cardiac progenitor cells (CPCs). These two populations of stem/progenitor cells are nested within the myocardium in different states: (1) quiescent and undifferentiated stem cells that, upon stimulation, are activated becoming cycling cells to expand the tissue-specific stem cell compartment of the heart [50–52] and (2) cycling progenitor cells that become committed expressing the transcription factor characteristic of one cardiac cell lineage to generate a large progeny of differentiated cells [52–54] (Figure 1). CSCs are indeed clonogenic, self-renewing, and multipotent, giving rise to a minimum of three different cardiogenic cell lineages (myocytes, smooth muscle cells, and endothelial cells) both *in vitro* and *in vivo*, and exhibit significant cardiac tissue regenerative capacity [54–56] (Figures 1 and 2). These cells, identified in particular for being c-kit positive and negative for blood/endothelial markers (Lin^−^ckit^pos^ CSCs) [55], express also different and well-characterized membrane markers (Sca-1, Abcg-2, Flk-1, CD105, CD166, and PDGFR-*α*) and transcription factors (Isl-1, Tert, Bmi-1, Gata-4, Mef2c, Nkx2.5, and Wt-1) [21, 55, 57–61]. Importantly, we recently demonstrated that a proper and physiological c-kit expression in the CSC population is necessary for their activation and survival and their cardiomyogenic differentiation potential *in vitro* and *in vivo* and that a gain-of-function mutation in the c-kit kinase domain increases CSC's myogenic and angiogenic potential *in vitro* and *in vivo* [55, 56, 62, 63]. On the other hand, CPCs are immature but already committed myocardial cells that can proliferate and mature into its respective precursor which, in turn, develops into one of the main cardiac cell lineages. Thus, CSCs/CPCs are actively involved with cardiomyocyte turnover, preserving myocyte number, attenuating the accumulation of hypertrophied CMs, and increasing the number of newly formed cardiac cells. We repeatedly and reproducibly demonstrated that in response to different and diffuse cardiac injuries, endogenous CSCs became activated to reenter the cell cycle, multiplying themselves to progress and acquire cardiac lineage commitment and finally differentiate into new CMs among other cardiac cell types [44, 55, 56, 62, 63] (Figure 2). Our results provide the indisputable evidence that CSCs possess cardiomyogenic potential as it would be expected from true cardiac-specific stem/progenitor cells. This conclusion is further strengthened by the normal structural organization and functional activity of CMs generated *in vivo* in response to CSC transplantation after cardiac injury in rodents [44, 55, 62, 63].

CSC-based therapies have been then recently the focus of most of the preclinical and clinical studies in regenerative cardiology becoming however the most highly debated topic of cardiac regeneration. Thus, to fully elucidate the biology of CSCs, including their senescent phenotype with age, is pivotal to develop future reliable approaches in order to manipulate and/or to therapeutically target them *in vivo* to enhance and improve their myogenic differentiation potential for cardiac tissue maintenance or repair in humans.

## 3. The Impact of Ageing on Endogenous CSCs: A Theoretical Everlasting Lifespan Limited in Reality by Cellular Senescence

If cardiac homeostasis is dependent on cell regeneration from the activation of resident endogenous CSCs, it can be predicted that the loss of CSC function, either as a consequence of their death or because they become over time nonproductive, should result in a progressive decrease in tissue homeostasis [64]. This hypothesis has been tested to explain the inevitable decompensation that occurs with age in the heart of simple and more complex organisms producing an impairment in cell homeostasis and a decline in its regenerative capacity, a process that limits lifespan [37, 65]. It has been indeed postulated that during aging, the primitive CSC pool undergoes depletion and/or attenuation in proliferation, and as a result, the heart evolves an inadequate regenerative response which contributes to the development of the aging cardiomyopathy [37, 52]. Thus, the critical question is whether aging-associated events directly affect the CSC pool and whether chronological age impacts on the number and properties of CSCs.

The ability of stem cells to self-renew, as well as their capacity to differentiate into single or multiple lineages, is regulated by specific signalling and by their specific niche organization [66, 67]. Thus, in the past years, many studies have focused the attention on the importance of the tissue microenvironment to preserve the primitive pool of CSCs in the healthy heart [52]. The niche microenvironment protects stem cells from damaging stimuli [52, 68–70] maintaining their function in tissue homeostasis [71]. Age-associated disorders in the myocardium are linked to changes in the cardiac microenvironment and an impaired or dysregulated communication between the aged microenvironment and the resident stem/progenitor cells that leads to a missing activation in response to injury [72]. As a consequence, there are changes in tissue maintenance whereby, in the absence of their natural milieu, CSCs cannot preserve their stemness potential and acquire a high probability to undergo a nonreturn process that leads to the exhaustion of their compartment [52].

Thus, it has been postulated that a mitotic clock regulates CSC turnover and their lifespan [73] and that this phenomenon leads to the development of the aging cardiac myopathy because cardiac tissue homeostasis changes in function of the accumulation of older and senescent CSCs [36, 74, 75]. Senescent cells are characterised by an altered gene expression profile and epigenetic modifications as well as an altered secretome with release of components which act on adjacent and distant cells causing fibrosis and inflammation [76–81]. The latter two disorders further impair the adult myocardium as well as its ability to respond adequately to the increases in physiological loads due to cardiomyocyte number progressive attrition.

Multiple markers have been utilized for the identification of senescent CSCs/CPCs and cardiomyocytes *in vitro* and *in vivo*. In an interesting and elegant work, Castaldi and colleagues [33] have shown how ageing affects the functional properties of CSCs comparing c-kit+ CPCs isolated from 3- and 24-month-old wild-type (WT) mice. CPCs isolated from aged mice display a diminished proliferation rate and stemness potential and differ from those of young mice in morphology and expression of molecular markers of senescence. CPCs isolated from aged mice present a flattened morphology accompanied by increased SA-*β*-Gal and p16 mRNA expression, a dramatic downregulation of the stemness marker LIN28, and a deficit in cardiac differentiation potential [33]. We and others have shown that CSCs/CPCs upregulate cardiac lineage markers in response to dexamethasone (Dex) treatment *in vitro* and that the TGF-*β*/Wnt/*β*-catenin pathways are instrumental to promote CSC differentiation, expansion, and survival [55, 82–85]. Based on this evidence, Castaldi and colleagues [33] have compared the response of young and aged CPCs to Dex treatment, showing that the aged CPCs fail to upregulate cardiac lineage markers when compared to the young counterpart. They also investigated the mitochondrial activity of CPCs showing that aged CPCs fail to activate mitochondrial biogenesis and display an increased expression of proteins involved with oxidative phosphorylation in response to Dex treatment. Moreover, they found that the *β*-catenin and TGF-*β* gene expression is upregulated in young CPCs in response to Dex treatment whereas these responses are completely lost in aged CPCs.

**Figure 3:**
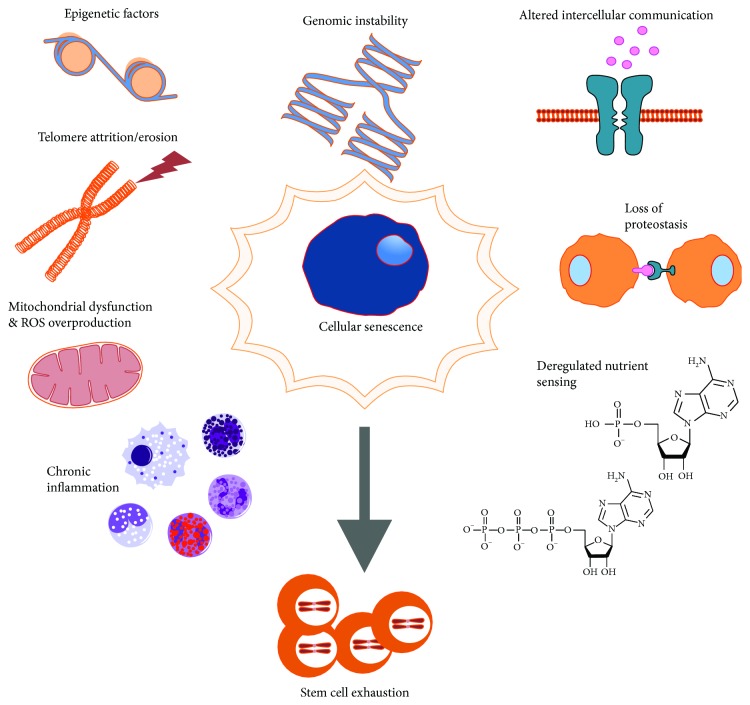
Schematic representation of the mechanisms implicated with adult stem cell senescence owing to tissue-specific stem/progenitor cell exhaustion in aging.

The occurrence of senescence markers in cardiac cells has been explained by two main mechanisms: one telomere dependent and the other telomere independent (Figure 3). The telomere-dependent mechanism is a cell cycle arrest characterized by a process called telomere shortening [36, 37, 65, 86, 87]. In particular, the ability of CSCs to produce an infinite number of divisions is a mechanism tightly controlled by telomeres and telomerase [88, 89]. The latter restores telomere length after each cell replication preventing the replicative senescence limit. In most adult tissues, telomerase activity has been found insufficient to compensate the progressive telomere attrition that occurs with aging [90]. Taking advantage of the relatively short mouse lifespan (despite very long telomeres), genetically modified mouse models have been used to study CSC activity during ageing [91–93]. Using a telomerase-null mice model, it has been reported that the progressive loss of telomere sequences leads to the loss of organism viability after 3-6 generations [89]. Moreover, telomerase-null mice display a profound attenuation in new CM formation and an increased apoptosis and hypertrophy of the remaining CMs [89]. The telomere shortening process leads overtime to genomic instability, characterized by the expression of senescence markers like the tumor suppressor p16INK4a [89, 94–96]. p16INK4a expression is extremely low or practically undetectable in intact viable young cells and tissues but becomes readily apparent in cells induced to senescence. This protein maintains the tumor suppressor Rb in a hypophosphorylated active state inducing cell cycle arrest [97] or initiating apoptosis by increasing p53 expression via downregulation of Mdm2 [98]. The progressive accumulation of p16INK4a-positive cells within the aged tissue *in vivo* [37] might have a damaging influence over neighbouring cells. Baker and colleagues [79] have indeed demonstrated, using a transgenic mouse model characterized by premature tissue senescence and by a markedly shortened lifespan, that the inducible elimination of p16INK4a-positive senescent cells delays the ageing-associated disorders in this mouse model. Thus, it is expected that the manipulation of the tissue microenvironment and homeostasis could delay or even revert CSC dysfunction associated with age.

To assess whether aging of endogenous CSCs is ruled by an internal cell clock or by a response to the environmental milieu, we studied the natural aging of CSCs and its effect on their growth and differentiation in WT mice as compared to transgenic mice (TG) with CM overexpression of the insulin-like growth factor-1 (IGF-1) [37]. The expression of IGF-1 receptors and the synthesis of IGF-1 have been found attenuated in aged CSCs [37]. The IGF-1/IGF-1 receptor induces CSC division, upregulates telomerase activity, maintains telomere length, hinders replicative senescence, and preserves the population of functionally competent CSCs. The IGF-1 pathway has been implicated as a mediator of CSC senescence whereas increased IGF-1 signalling attenuates ageing-associated markers [37]. At 22 months, ≥70% of CSCs in WT mice, but only ~10% in the TG mice, are p16INK4a positive, indicating an increased CSC senescence and a block in the cell cycle with age. At 22 months, CSC apoptosis is 5-fold higher in WT mice when compared with TG mice. Concurrently, the decrease in CM number and cardiac failure in the WT old versus young mice is absent in old TG mice that show a normal CM number and a normal ventricular performance as compared to their young counterparts. Therefore, this data provides the proof of concept that with age, senescence of endogenous CSCs is not regulated by an internal cell or organismal clock but could be modulated by the microenvironment with important physiological consequences. These evidences suggest that the aged phenotype in the CSC population is not irreversible because CSCs can be rejuvenated and returned to a normal proactive function in response to appropriate cardiopoietic growth factors [37].

## 4. Free Radical Theory of Aging: ROS-Dependent Senescence of Endogenous CSCs

Despite that the loss of telomere/telomerase integrity is a major determinant of tissue and organism alteration during the years [89], aging is mainly caused overtime by a reduced cellular capacity to respond to stress [99, 100]. Indeed, the telomere-independent cellular aging, or stress-induced premature cellular senescence, is promoted by oxidative stress and/or genetic/epigenetic alterations and oncogene activation [81, 101–106] (Figure 3).

Nowadays, it has been largely documented that with the increase of the organismal age and with the occurrence of cardiac diseases, there is a concomitant increase in the oxidative stress within the tissues [107, 108]. ROS are chemically reactive molecules including free radicals, oxygen ions, and peroxides, generated through a variety of extracellular and intracellular events, in particular by oxidative phosphorylation in the aerobic metabolism starting from molecular oxygen. ROS activity and concentration are finely controlled by antioxidant molecules [109]. When excessively produced and free radical scavenging systems are depleted, ROS can damage macromolecules [110], affecting cell proliferation and causing the so-called oxidative stress, defined as a change in the balance between oxidant and antioxidant elements [111]. The cardiac work overload that occurs with aging produces oxidative stress leading to genomic instability and cellular senescence or apoptosis; oxidative stress subsequently contributes to the development and/or progression of different diseases including heart failure [112–115].

ROS can be considered as signalling molecules playing a major role in the crosstalk between metabolism and stem cell fate decisions as well as pivotal factors regulating self-renewal, differentiation, and senescence of stem cells [37, 116, 117]. Thus, it is possible that high levels of ROS may lead to several changes in CSC properties such as partial depletion of the primitive pool, loss of self-renewing capacity, increased symmetric division with formation of daughter committed cells, impaired ability to migrate, and forced entry into a senescent state. The healthy behaviour and the efficient regenerative response of the myocardium to wear and tear and damaging stimuli have been correlated to the low hemodynamic stress that seems to persist within the CSC microenvironment, together with O2 gradient within the tissue [36, 52, 118]. Indeed, similarly to embryonic stem cells during foetal life, the long-term preservation of the adult CSC compartment requires a hypoxic milieu in which CSCs are stored in a quiescent state. The physiological normoxia is necessary for the activation of CM commitment, and it leads progressively with aging to CSC senescence and its pool exhaustion [36, 52, 118]. ROS may condition the balance between hypoxia and normoxia in the aged myocardium, influencing the characteristics of the surrounding milieu of the stem/progenitor cells leading to telomere erosion, formation of dysfunctional CMs, and a decline in tissue regeneration processes [64]. For example, the renin-angiotensin aldosterone system (RAAS) has been found linked to cardiovascular disease and age-related declines in cardiac function [119, 120]. The accumulation of Ang II contributes during aging to chromatin remodelling, DNA damage, and gene expression activation together with telomere attrition/shortening, uncapping, and expression of p16INK4a and p53 at a cellular level. As cited above, all these events have been associated to cellular senescence and death [121–125]. Thus, the balance between hypoxic and normoxic conditions in the CSC compartment is critical for cardiac homeostasis and for the preservation of the CSC pool during the lifespan to sustain cell turnover and the demands of the myocardium. Indeed, a decrease in ROS levels may improve stem cell maintenance [126, 127], suggesting that antioxidant enzymes could play a significant role in the preservation of CSC regenerative function [128].

Overall, while a link between metabolic rate and the rate of aging has been provided, it is less clear whether and how mitochondrial metabolism and ROS are implicated in CSC aging.

## 5. Evidence for Epigenetic Changes and the Role of Noncoding RNAs in Cardiac Aging

As above discussed, cardiac aging in mammals can be defined by definite hallmarks: telomere attrition, mitochondrial dysfunction, cellular senescence, stem cell exhaustion, genomic instability, altered intercellular communication, loss of proteostasis, and deregulated nutrient sensing [78, 129, 130] (Figure 3). Among these alterations, a significant effort has been recently made in the understanding of the epigenetic changes leading to aging-related diseases. With aging, there is an increased transcriptional noise in which a stochastic deregulation of gene expression leads to increased DNA damage and cellular degeneration and death [131]. Experiments on transcriptional profiling of single CMs isolated from old mice showed considerable variation in transcript levels of a panel of heart-specific housekeeping genes compared to those from young controls [131]. Nongenetic contributions, which are the alterations secondary to environmental stimuli or nutrient availability, play a major role in longevity. Continuous interactions among genetic/epigenetic and environmental/stochastic factors influence the possibility to reach the limit of the human lifespan. Calorie restriction [132], lowering of basal metabolic rate [133], upregulated stress response [134], and reduced fertility [135] have been shown to correlate with lifespan extension and to influence the oxidative stress level. Nevertheless, accumulating evidences have revealed that genetic differences and somatic mutations also underlie longevity, providing a link between aging and genetic and epigenetic mutations [136]. Epigenetic changes, leading to impaired biochemical pathways and genetic processes [137, 138], have been found to be associated with cardiac dysfunction and aberrant cardiac regeneration [139–141]. Furthermore, studies conducted to further the understanding of the molecular mechanisms underlying exceptional human longevity also suggest an important genetic component [142, 143]. In an interesting study conducted in centenarians, a genome-wide methylation analysis has identified genes that are hypo- or hypermethylated during ageing when compared to those in young people [144]. Accordingly, genetic/epigenetic variations may predispose cells to better adapt to cellular stress and to better survive an adverse environment.

Epigenetic alteration is a heritable alteration in gene expression or cellular phenotype in organs and tissues in which DNA, RNA, and proteins are chemically or structurally modified, without changes in their primary sequence. This phenomenon produces chromatin alteration and influences gene expression and cell division by regulating access of the transcriptional machinery to DNA [145]. A large number of epigenetic alterations occurring with age have been demonstrated and well characterized and include DNA methylation and hydroxymethylation, histone modification, chromatin remodelling, RNA methylation, and the regulation by small and long noncoding RNAs [146–149]. From yeast to humans, an emerging epigenetic mark in aging cells is the general loss of histones, which is linked to cell division [150, 151]. Despite that the loss of histones is the major determinant of epigenetic alterations, additional important modifications that occur at a chromatin level are histone methylation and acetylation [152]. With age, mammalian cells undergo global DNA hypomethylation and local DNA hypermethylation [153], a pattern which fits with a global heterochromatin deregulation and modification influencing stress tolerance and/or modulating specific pathways. Many histone modifications are involved in activation or suppression of genes influencing lifespan. In addition, the age-dependent altered expression of chromatin-modifying enzymes could induce epigenetic changes in adult stem cells resulting in their decline and leading to senescence [154]. A chromatin-related senescence phenotype has been found in aged tissues from various organisms, but the real mechanisms underlying the connection between senescence and longevity are unclear.

Epigenetic and epigenomic modifications occurring during aging also dictate stem cell fate. As a typical example, in the hematopoietic system, the epigenomic modifications enforce increased self-renewal and decreased differentiation [155]. The functional potential of hematopoietic stem cells (HSCs) declines during aging, and it contributes to hematopoietic pathophysiology in the elderly and loss of the hematopoietic cell pool [156, 157]. Site-specific alterations of DNA methylation occur at genomic regions associated with the hematopoietic lineage potential and at target genes expressed in downstream progenitor and effector cells. Young HSCs gain DNA methylation on regions associated with nonhematopoietic lineages, whereas the transition to old age is marked by gains of DNA methylation at genomic regions associated with the lymphoid and erythroid lineages that restrict the potential of old HSCs [156, 157]. In contrast to young adult HSCs, which showed robust activity in transplantation assays with comparable lineage predisposition and reconstituting potential, HSCs from old mice showed diminished repopulating activity lineage potential [156–160]. Beerman and colleagues [161] have found that the replicative decline in HSC and DNA methylation is largely dependent on the proliferative history of HSCs in a process that appears to be telomere independent. The evidence that HSC self-renewal is limited by replicative history may explain why the loss of HSC quiescence or sustained cycling may lead to premature HSC exhaustion [162]. Thus, it is possible to speculate that this evidence could explain the cell exhaustion that has been shown to exist also in the CSC compartment with age [52, 162]. Unfortunately, the epigenetic signature of CPCs is not clearly known, although the epigenetic mechanisms underlying differentiation of CSCs during development of the heart have been well documented [163]. The plasticity and reversibility of DNA methylation opens the possibility of therapeutically targeting its regulators to restore the function to aged stem cells. Nowadays, epigenetic aging research is trying to characterize key epigenetic steps leading to aging-specific signalling pathway modification and genetic variants with the aim to restore them using genetic manipulations, uncovering specific enzymes for targeted therapeutic strategies to improve lifespan and health span.

Among the environmental and epigenetic factors, there are a substantial variety of different RNAs that are actively transcribed from the human genome and have been reported to be implicated in transcriptional regulation, posttranscriptional gene control, epigenetic control, nuclear genome organization, and onset of diseases related to aging [164–167]. Recently, long noncoding (lnc) RNA has emerged as a crucial class of regulatory molecules responsible for specialized biological processes during cardiac development, pluripotency, cell fate determination, homeostasis, and disease [168, 169]. Cardiomyopathy has been associated with a reduced expression of Mhrt which is a lncRNA that targets the chromatin-modifying protein complexes [170]. Up to now, the role of lncRNAs in cardiac cells and in the ageing of cardiac tissue has not been specifically investigated. However, a number of lncRNAs have been identified to be important in the processes involved with ageing such us cellular senescence that in cardiomyocytes is associated with multiple cellular processes, for example, ROS production by mitochondria [171].

Abdelmohsen and colleagues [172] used RNA sequencing to compare transcripts expressed in proliferating cells and terminally arrested cells identifying lncRNAs involved in cellular senescence [172]. They found Malat1, one of the most widely studied lncRNAs involved in cell cycle arrest, downregulated in senescent cells. Knockdown of Malat1 blocks the progression of the cell cycle at the G1/S phase, resulting in more senescence-like cells [173]. We already discussed as telomere erosion causes telomere shortening upon each cell cycle and results in senescent cells. The lncRNA telomeric repeat-containing RNA, TERRA, can base pair with the RNA template of telomerase and can also bind to the telomerase reverse transcriptase polypeptide to inhibit telomerase activity [174, 175]. As a result, cells expressing increased levels of TERRA become senescent [174, 175]. Interestingly, the structure and function of TERRA are conserved among eukaryotes, rendering TERRA-mediated regulation of telomerase a promising therapeutic strategy against cancer and age-associated diseases.

Xia and colleagues have demonstrated that mesenchymal stem cells (MSCs) isolated from aged mice display a reduced proliferation and paracrine signalling, an increased oxidative stress, and expression of lncRNA-p21 compared with MSCs from younger mice. Silencing lncRNA-p21 in aged MSCs using small interfering RNA (siRNA) enhances cell growth and paracrine function and decreased oxidative stress through Wnt/*β*-catenin pathway modulation [176]. The regulatory effects of noncoding RNA molecules might be therefore used to improve cardiac progenitor cell proliferation, cardiac cell differentiation, cardiac reprogramming, and cardiac survival.

Accordingly, a large number of studies have focused their attention principally on the possibility to modify stem cells, in order to control their senescence or to activate their differentiation potential using RNA molecules that directly act on DNA by inducing epigenetic changes. As shown in MSCs, histone deacetylase inhibitors produce an increase in the acetylation of histones adjacent to the coding regions of microRNAs, which engage in MSC aging [177]. MicroRNAs expression changes during life-time and modulates the senescence of adult stem cells by targeting genes involved in DNA damage, epigenetic changes and metabolism. At the same time, epigenetic mechanisms may regulate an enormous number of microRNAs, mainly by DNA methylation that represses gene activity by preventing the binding of transcription factors to gene promoters or by favouring the recruitment of chromatin-modifying enzymes [178]. Studies of their expression and targets have the potential to supply critical information on the aging process and its propagation through cells, tissues, and organs [179]. The detection of several circulating RNAs in biological samples has been correlated with different ages and some of them have been associated with aging myopathy and epigenetic mechanism underlying development and progression of cardiovascular diseases [139, 180]. Thus, a combination of specific circulating lncRNAs and microRNAs may be a precious tool to estimate the age-related deterioration of different organs and age-related CVDs. Consequently, these RNA molecules have been proposed as diagnostic/prognostic biomarkers and therapeutic targets.

Since DNA methylation is intimately involved with diseases and histone modification, it is not unreasonable to suggest that the post-translational modifications of histones may also be vital factors governing cell cycle, apoptosis, and response to environmental cues [181]. Methylation of microRNA promoters may be pivotal to determining the ability of microRNA transcripts to be successfully produced. Dysregulation of this event may induce the loss of functional microRNAs and pathological developments in tissues or cellular senescence [182]. Indeed, microRNAs are epigenetic regulators of gene expression playing an essential role in the post-transcriptional regulation and suppression or expression of many target genes [183]. For example, microRNAs have been linked with the age-associated hypertrophic growth of CMs, a typical feature of cardiac cell remodelling in response to stresses [184]. CM hypertrophy leads molecular and morphological changes, including altered expression of *α*-MHC, *β*-MHC, ANF and SERCA2, producing maladaptive cardiac hypertrophy and finally cardiac decompensation [184]. The reactivation of this set of cardiac genes has been found to be regulated by microRNAs.

It has been shown that aging influences circulating levels of some microRNAs in animal models of physiological aging as well in elderly subjects compared to younger ones [185, 186]. A large number of microRNAs have been described to be differently expressed and to regulate different cell types and pathways during cardiac aging [187]. Among the many microRNAs upregulated with cardiac aging, there is miR-21 [187] that has a profibrotic effect and is highly expressed in cardiac fibroblasts during injury. In addition, overexpression of Ago1 and Ago2 synergistically induced miR-21 and miR-21^∗^, suggesting a regulatory role for Ago proteins [187]. Another microRNA involved in cardiac aging and expressed in aged CMs is miR-22 [188, 189]. With age, the expression of miR-22 has been found increased in the cardiac tissue [190]. miR-22 overexpression is able to inhibit cell cycle progression and is sufficient to provoke CM hypertrophy, and it may accelerate cardiac fibroblast senescence [188]. The inhibition of miR-22 stimulates cardiac autophagy, prevents maladaptive remodelling, and enhances cardiac function postmyocardial infarction in older mice, but not in younger ones [189].

Important evidences suggest a crucial role of microRNA in CSCs too. miR-34 is an important regulator of senescence through its role in several pathways that include cell cycle, telomere shortening, and DNA damage response [190, 191]. The *in vivo* silencing of miR-34a by injection of antisense oligonucleotides (antagomirs) can partially rescue the cardiac aging phenotypes in mice and promotes cardiac progenitor cell growth rate [190, 192]. Thus, the rescue of cardiac repair activity through miR-34 inhibition could be used for human heart disease treatment [192]. This finding supports the potential of gene therapy to reverse cardiac aging using microRNAs. However, as one microRNA may have multiple targets, gene therapy targeting microRNA may trigger undesirable side effects. An alternative approach is to identify the microRNA targets that mediate cardiac aging responses and manipulate the specific target genes as treatment strategy.

## 6. CSC Rejuvenation: Intrinsic and Extrinsic Molecular Manipulation to Revert CSC Aging

All the evidences above detailed show that aging and cellular senescence are a major hindrance to the endogenous regenerative efficacy of CSCs/CPCs. However, the persistence in aged decompensated hearts of a population of functionally competent CSCs with long telomeres [193] generates the hypothesis that endogenous CSCs may be indeed rejuvenated to regain robust regenerative potential. This population of functional yet old CSCs lacks senescent markers, expresses telomerase and cycling proteins, such as Ki67 [35, 36], and displays the capacity to migrate to injured zones generating a healthy progeny of young CMs. Indeed, as demonstrated in old humans, a pool of CSCs dividing by asymmetric chromatid segregation seems to maintain a growth reserve and self-renewing potential in the cardiac tissue, critical variables for effective cardiac homeostasis and repair during aging [193]. This evidence may correlate to epigenetic modifications in the longer telomeres of human CSCs with old intact DNA that may account for the more effective maintenance of chromosomal ends in this CSC pool [193, 194]. These evidences postulate that nonsenescent and undamaged CSCs in the heart may be the target of regenerative therapies to improve cardiac performance with prolongation and improvement of lifespan in the elderly [36].

On the other hand, it is expected that a better understanding of the metabolic pathways and molecular mechanisms active in adult stem cells in old tissues may be helpful to develop genetic approaches or drugs to preserve their stemness potential during aging and to manipulate their quiescence, self-renewal, and differentiation [2, 195]. Several strategies able to decrease ROS levels, to restore or increase telomerase activity and telomere length in order to delay the natural aging process of the entire organism, have been studied in the past decade. For example, mouse models of telomere deficiency show an increase in age-related cardiac hypertrophy and decompensation [100, 196], while mice overexpressing telomerase present a nonpathologic cardiac hypertrophy and a resistance to the acute ischemic insult from myocardial infarction [197], demonstrating the importance of the telomere-telomerase system in cardiac cell aging studies in order to promote their rejuvenation. Through its cell cycle inhibition effects, p16INK4A is the major regulator of self-renewal of various adult stem cell types playing a role in the decreased efficacy of stem cell proliferation [198, 199]; it has been therefore the target of many aging studies. Cells genetically modified to overexpress p16INK4A display a growth arrest similarly observed in senescent cells that typically show an elevated p16INK4A expression [200, 201]. As cited above, its upregulation can be induced by stress stimuli such as DNA damage and oxygen radicals [199]. Since several studies have shown that p16INK4A silencing may significantly delay cellular entry into senescence [79, 202], Khatiwala and colleagues [203] have knockdown this gene as a method to attempt rejuvenation of aged human CPCs in order to promote myocardial repair. They demonstrated that knockdown of p16INK4A reverses the senescent phenotype and has a beneficial antioxidant effect on aged human CPCs with a decrease of ROS by approximately 50%. Because of ROS's role in the acceleration of cellular senescence, knockdown of the p16INK4A gene may exert beneficial effects in extending lifespan inducing cellular rejuvenation of the aged human CPCs [203].

Bmi-1 is a member of the Polycomb repressor complex 1 that mediates gene silencing by regulating the chromatin structure and is indispensable for self-renewal of both normal and cancer stem cells [204, 205]. Bmi-1 controls self-renewal and cell cycle by regulating the tumor suppressor proteins p16Ink4a and p14Arf [204, 205]. Furthermore, it has been shown that Bmi-1 prevents adult stem cell aging, at least partly, by blocking expression of the cyclin-dependent kinase inhibitor p16Ink4a [204, 205]. Additionally, Bmi-1 inhibits ROS-induced oxidative DNA damage along with facilitating the overall DNA damage response, contributing to maintaining genome integrity and resistance to genotoxic therapeutic reagents. Therefore, dysregulation of the Bmi-1/p16Ink4a pathway is considered key to the loss of tissue homeostasis and development of associated degenerative diseases during aging. Not surprisingly, Bmi-1 is also expressed in the hCSCs [118]. Importantly, the undifferentiated hCSCs from old donors exhibit a lower level expression of Bmi-1 when compared to hCSCs from young donors. hCSCs infected with a lentivirus constitutively expressing Bmi-1, increased significantly the cloning efficiency and BrdU incorporation of cloned hCSCs. Conversely, downregulation of Bmi-1 with specific si-RNA significantly impaired cloning efficiency and proliferation of the hCSCs, while increasing the expression of p16Ink4a (our preliminary data).

Despite that, during the late adulthood, the heart maintains a functionally competent CSC compartment, the aged cardiac phenotype produces an accumulation of senescent CSCs. Many evidences have pointed to the possibility to revert the senescent cardiac phenotype using growth factors or cardioprotective molecules, fostering CSC physiological turnover in order to maintain their stemness potential. IGF-1 [37, 206], Pim-1 [91], Follistatin-like 1 [207, 208], and growth differentiation factor 11 [208–210] are all together examples of antiageing factors capable to induce endogenous regeneration in the aged heart.

The consequence of oxidative stress in the average lifespan of CSCs may be reverted by the administration of IGF-1. This growth factor not only produces a decrease in the oxidative stress in the cardiac tissue [37] but is able to act on DNA damage repair by homologous recombination [211]. Moreover, administration of IGF-1 in aged mice models enhances phospho-Akt expression and the telomerase pathway, delaying cellular aging and death [37]. A reduced phospho-Akt expression has been associated with ageing and is now thought to act as a main modulator of telomerase activity in the survival molecular signalling. Thus, therapies aimed at stimulating Akt expression have been shown to bypass some ageing effects [37]. The redox effector protein-1 (Ref-1) is another important protein that plays an essential role in DNA repair and redox regulation of several transcription factors. Particularly, Ref-1 seems to maintain the redox status and survival of adult CSCs. Gurusamy and colleagues [4] have shown that Ref-1 inhibition robustly induces the increase of intracellular ROS and the treatment of CSCs with a low concentration of H2O2 seems to induce Ref-1-mediated survival signalling through phosphorylation of Akt. They have also shown that after H2O2 treatment in Ref-1-inibited CSCs, the expression of cardiac differentiation markers (Nkx2.5, MEF2C, and GATA4 and *α*-sarcomeric actinin) was significantly elevated. Further, Ref-1 inhibition seems to produce in CSCs an increased p53 expression leading to cell death and a decreased Akt phosphorylation. Finally, Gurusamy and colleagues demonstrated that treatment with the ROS scavenger N-acetyl-L-cysteine attenuates oxidative phosphorylation and cardiac differentiation. Thus, Ref-1 plays an important role in maintaining the redox status of CSCs and protects them from oxidative injury-mediated cell death and differentiation [4].

Another promising study is focused on the ex vivo modification of CPCs using Pim-1 kinase for its potential to alleviate cellular senescent characteristics. Mohsin and colleagues [212] have used a lentivirus to overexpress Pim-1 in CPCs isolated from a 68-year-old heart failure patient. The Pim-1-modified CPCs have been transplanted into an immunocompromised mouse model of myocardial infarction showing enhanced persistence, survival, proliferation, and differentiation potential compared with the older nonmodified counterpart. Pim-1 is cardioprotective and able to rejuvenate CSC phenotypic and their functional properties, restoring youthful telomeric length, enhancing replicative capacity, and decreasing the levels of p16Ink4a and p53 [212]. Concurrently, another elegant study provided the evidence that nucleostemin (NS) expression is lower in CPCs isolated from aged compared to young mice. Hariharan and colleagues [213] demonstrated that aged CPCs engineered for NS expression preserved their stemness properties while deficiency of NS led to myocardial ageing due to telomere shortening.

The ability to rejuvenate human cardiac progenitor cells ex vivo by Pim-1 or NS modification is encouraging proofs of concept for the combination of gene and stem cell therapies to develop efficient CSC rejuvenation in order to prevent and/or treat myocardial decompensation as a result of cardiac aging.

## 7. Future Clinical Perspectives to Delay Cardiac Aging Rejuvenating the Senescent Human Heart

The average lifespan of the human population is increasing. Since the vast majority of all the worldwide deaths occur for cardiovascular diseases in a segment of population older than 70 years, the prevalence of cardiovascular pathology makes heart disease largely an elderly disease.

The aged myocardial tissue is characterized by several pathological or physiological conditions including increased ROS production and oxidative stress, loss of telomere/telomerase integrity, and telomere shortening (Figure 3). Together, these processes contribute to the development of a senescent phenotype in cardiac cells ensuing in heart tissue homeostasis abnormalities leading to diastolic and systolic dysfunctions in the aged myocardium. Cardiac tissue homeostasis is mainly impaired by the accumulation in the extracellular matrix of inflammatory cytokines which negatively influences the behaviour of neighbouring cells. This phenomenon compromises CSC function during aging leading to an inadequate regenerative response and cardiac decompensation. Concurrently, cellular lifespan may depend on a mitotic clock and this phenomenon may have a pivotal role in cardiac aging and in the attenuation of cell turnover in the old myocardial tissue. Moreover, aging represents a decline in cardioprotective systems affecting pathophysiological cardiovascular pathways, a process that is a fertile ground for the development of cardiomyopathy. As consequence, a better insight into the mechanisms of cardiac aging could lead to discover factors implicated in cardiac pathophysiology on one side and on the other to improve prevention strategies for aging-related human cardiovascular diseases.

As summarized in this review, nowadays, cardiovascular research produced a significant number of studies displaying methodologies and therapeutic approaches to delay or treat cardiac aging. These approaches could be able to decrease ROS levels, as Gurusamy and colleagues [4] shown using Ref-1, or may include gene therapy to modify the levels of expression of several genes and proteins involved in cardiac aging (i.e., p16INK4A, Bmi-1, and Akt). Despite that future studies are required to evaluate the translational potentials of cardiac stem cell therapy, the possibility to manipulate these cells to express growth factors or cardioprotective molecules (i.e., IGF-1, Pim-1, Follistatin-like 1, and nucleostemin) may become an important goal to develop strategies to fostering autologous CSC physiological turnover in order to maintain their stemness potential, to rejuvenate them, and to improve their beneficial regenerative/reparative properties.

Interestingly, centenarians may become a model of successful ageing because they are less susceptible to cardiovascular diseases and all ageing-related diseases [142, 214, 215]. Villa and colleagues have measured the serum levels of BPIFB4 protein in subjects able to reach extreme ages and in long-living individuals (LLIs) [216]. They identified a variant in the BPIFB4 transcript downregulated during aging [217]. Thus, the serum levels of BPIFB4 could correlate with the ability to reach extreme ages and with LLI health status [216]. BPIFB4 appears to have a beneficial effect on cellular homeostasis, where its overexpression activates stress response (with upregulation of heat shock proteins), and proteostasis (especially involved in genomic integrity and activation of protein synthesis). On the other hand, the possibility to study CSCs belonging to centenarians may become relevant to detect hypothetical different molecular mechanisms and biological characteristics and compare them with our current knowledge about CSCs. These studies may become a precious source to definitively understand how to delay cardiac aging rejuvenating the senescent human heart.

Overall, as most donors and in particular recipients in need of CSC therapy are of older age and exhibit a disease state, it is fundamental to acquire a better understanding of the biology and regenerative potential of the endogenous CSCs in order to design and develop better protocols and interventions for the regeneration of functional contractile mass following myocardial injury through the activation of these regenerative cells in situ.

## 8. Conclusions

**Figure 4:**
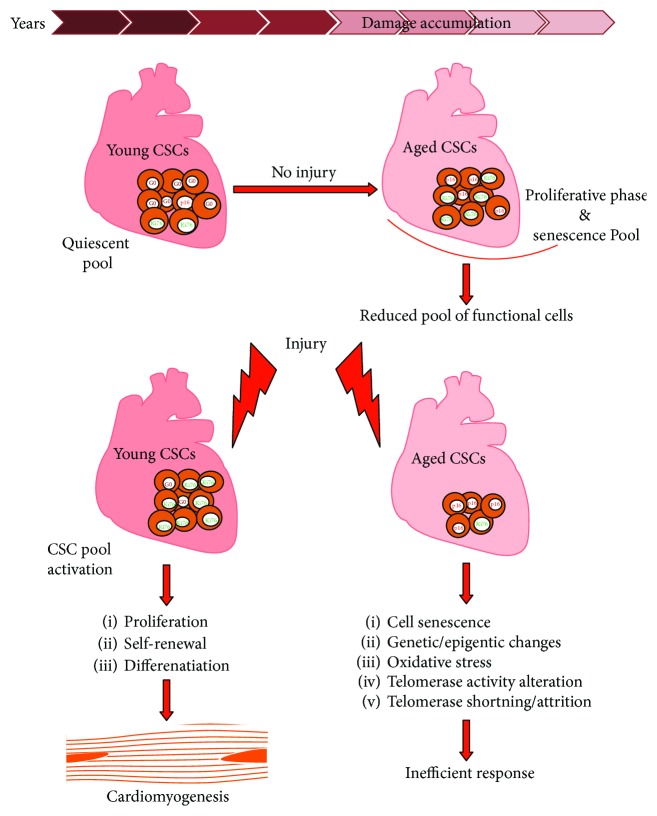
Schematic representation of the accumulation of “old” CSCs during cardiac homeostasis in aging and their impaired regenerative response after injury when compared to “healthy” young CSCs.

The present available evidence shows that the mammalian, including the human, myocardium possesses an “aged” CSC phenotype and this affects CSC self-renewal ability, differentiation, and regenerative potential (Figure 4). Thus, CSCs are not immortal. They undergo cellular aging in response to a variety of physiological and pathological demands. Nevertheless, the old myocardium preserves an endogenous functionally competent CSC cohort which appears to be resistant to the senescent phenotype occurring with aging. The latter envisions the phenomenon of CSC ageing as a result of a stochastic and therefore reversible cell autonomous process. Indeed, if CSC aging is really a stochastic cell autonomous process, then the possibility to rejuvenate the endogenous CSC population by stimulating their growth and self-renewing could be concrete. On the other hand, CSC aging could be a cell cycle-dependent process, affecting all or most of the endogenous CSC population, with a consequent irreversible loss of CSC regenerative capacity with time. If the latter is correct, it is predictable that the loss of CSC regenerative capacity with time progression is an inevitable phenomenon that cannot be rescued by stimulating their growth, which would only speed their progressive exhaustion. Moreover, to study the epigenetic signatures of healthy old people, i.e., centenarians, could be an important aspect to identify the role of chromatin modifications and plasticity in the longevity phenotype that seems to require a combination of stochastic and nonstochastic events to modulate genetic substrates leading to different outcomes [218]. It is possible that centenarians have a more powerful “engine” shaped by evolution and that environment, epigenetic system, and genetic predisposition could be components influencing their CSC pool preservation.

The determination of whether the aged phenotype of the CSCs is reversible or irreversible has importance for the future of myocardial regeneration. The majority of the data currently available predict that the “aged” CSCs can be rejuvenated and their properties reversed through manipulating certain intrinsic and extrinsic factors. Therefore, aged CSCs can be reintroduced back into the functional CSC pool. However, to reach this goal, several main questions remain to be answered: (a) Is the self-renewing potential of the cycling competent hCSCs isolated from an old heart similar or different from that of cycling hCSCs isolated from a young and healthy heart? What is the self-renewing potential of the clonal hCSCs isolated from these two types of hearts? Is the growth potential of cycling competent hCSCs from old hearts diminished? If so, is there an inverse correlation between cycling competent hCSC growth potential and the fraction of aged hCSCs in the tissue? (b) Is the quantitative and qualitative differentiation potential of cycling competent hCSCs constant or does it change as the fraction of aged hCSCs increases? (c) Is the diminished self-renewal and/or differentiation potential of the hCSCs from the old/failing hearts due to a diminished cycling or differentiation potential of these cells? Or, is it due to a change in their pattern of cell division, which increases the probability of producing a higher fraction of differentiated cells at the expense of the self-renewal of the hCSCs, as described below?

The answers to these and other similar questions will clearly rule the path toward a solid evidence-based clinical application of CSC rejuvenation strategies to prevent and treat cardiovascular diseases in the elderly.
